# Joint trajectories of sleep quality and cognitive function after acute ischemic stroke: a prospective cohort study using group-based dual trajectory modeling

**DOI:** 10.3389/fmed.2026.1809559

**Published:** 2026-06-26

**Authors:** Li Wang, Yuhan Cheng, Sibei Wan, Qian Wu, Yan Shi

**Affiliations:** 1 Department of Nursing, Shanghai Tenth People’s Hospital, School of Medicine, Tongji University, Shanghai, China; 2 School of Medicine, Tongji University, Shanghai, China

**Keywords:** acute ischemic stroke, cognitive function, group-based dual trajectory modeling, multinomial logistic regression, sleep quality

## Abstract

**Background:**

Sleep disorders and cognitive impairment commonly co-occur after acute ischemic stroke (AIS), yet their co-development is rarely modeled jointly.

**Objective:**

To determine whether sleep quality and cognitive function after AIS show asynchronous rather than automatically parallel recovery trajectories during the first year after discharge, and to identify distinct joint trajectory subgroups and baseline factors associated with trajectory membership.

**Methods:**

In this single-center prospective cohort in Shanghai (December 2023–October 2024), AIS inpatients were assessed within 24 h before discharge (T1) and at 3 (T2) and 12 months (T3). Sleep quality (PSQI; reverse-coded for modeling) and cognition (MoCA) were jointly modeled using group-based dual trajectory modeling (GBDTM). The optimal class solution was selected using information criteria and classification quality. Multinomial logistic regression (C1 reference) examined predictors of class membership. Generalized estimating equations (GEE) evaluated longitudinal changes in NIHSS, Barthel Index, IADL, and PHQ-9 across classes.

**Results:**

Among 515 participants, a 3-class solution was optimal: C1 sleep improvement–cognitive decline (29.51%, *n* = 152), C2 sleep decline–cognitive improvement (43.30%, *n* = 223), and C3 sleep improvement–cognitive improvement (27.18%, *n* = 140). For C2 (vs C1), widowhood, left-hemisphere infarction, and prior stroke history were associated with lower odds of class membership, whereas arrhythmia, atrial fibrillation, and baseline PSQI (T1) were associated with higher odds of class membership (all *p* < 0.05). For C3 versus C1, unmarried status was associated with higher odds of C3 membership, whereas left-hemisphere infarction, social medical insurance, and baseline PSQI were associated with lower odds of C3 membership. GEE showed no significant between-class differences in NIHSS, Barthel Index, or IADL at follow-up time points (all *p* > 0.05); PHQ-9 increased over time and was higher in C3 than C1 at T1 and T2.

**Conclusion:**

AIS survivors show heterogeneous and sometimes asynchronous joint sleep–cognition trajectories. Joint trajectory stratification and its correlates may inform targeted follow-up and individualized management.

## 1 Introduction

Acute ischemic stroke (AIS) is one of the leading causes of death and disability worldwide, with its morbidity and recurrence rates rising continuously, which significantly increases the loss of patients’ quality of life and the burden on health resources (1). In addition to motor, speech and other functional impairments, sleep disorders and cognitive impairment are common comorbidities after stroke, both of which are associated with rehabilitation outcomes, readmission and the risk of stroke recurrence (2). In current clinical management, sleep and cognition are typically assessed and intervened as relatively independent outcomes, respectively (3), whereas a study by Gottesman et al. (4) has indicated a bidirectional and dynamic coupling relationship between the two: poor sleep quality can affect attention, memory and executive functions through pathways such as neuroinflammation, circadian rhythm disturbance, activation of the hypothalamic–pituitary–adrenal axis and impaired brain network plasticity (5); meanwhile, cognitive impairment, along with its associated alterations in behavioral rhythms, reduced daytime activity and enhanced stress responses, may further disrupt sleep architecture and sleep maintenance (6). Therefore, the “sleep-cognition” axis after stroke is more likely to form an interactive dynamic system rather than a unidirectional causal chain (7).

Conceptually, sleep should be understood as a core neurobehavioral component of post-stroke recovery rather than as a secondary complaint. After stroke, sleep may influence recovery through several interrelated pathways, including circadian rhythm regulation, inflammatory activity, autonomic balance, emotional regulation, daytime activity, memory consolidation, and neural plasticity. Poor sleep may reduce rehabilitation engagement and impair attention, executive function, and learning efficiency, whereas cognitive impairment and emotional distress may further disrupt sleep maintenance and daily rhythms. Therefore, sleep quality is not only an outcome of post-stroke adaptation, but also a potentially modifiable recovery domain that may shape cognitive and functional recovery.

Nonetheless, the existing evidence is inconsistent in terms of the direction and strength of the association between sleep metrics (sleep quality, sleep duration or specific sleep disorders) and cognitive outcomes, suggesting significant evolutionary heterogeneity within the stroke population (8). More importantly, most previous studies have adopted cross-sectional analyses or conducted univariate longitudinal modeling of sleep and cognition separately, which makes it difficult to reveal their joint evolutionary patterns over time at the individual patient level (9). This thus limits the answers to clinically more meaningful stratification questions—for example, which patients present a composite high-risk trajectory of “persistently poor sleep accompanied by cognitive decline” (10), which patients exhibit a mismatched trajectory of “asynchronous changes in sleep and cognition,” and whether there are identifiable and intervenable predictors for these trajectories (11).

Against this backdrop, trajectory modeling offers a more appropriate methodological framework (12). Group-based trajectory model (GBTM) can identify distinct change subtypes and capture intra-population heterogeneity (13); on this basis, group-based dual trajectory model (GBDTM) is capable of simultaneously depicting the longitudinal changes in sleep and cognition within a single framework and identifying potential joint trajectory classes (14), thus serving the risk stratification and individualized intervention decision-making after stroke in a more direct manner (15).

Therefore, the central research question of this study was whether sleep quality and cognitive function after AIS recover synchronously or whether they follow asynchronous, non-parallel joint trajectories during the first year after discharge. In this study, sleep quality was conceptualized as a modifiable neurobehavioral recovery domain, rather than merely as a subjective symptom or secondary outcome. This conceptualization is based on the premise that sleep may both reflect post-stroke adaptation and actively shape cognitive recovery through circadian rhythm regulation, neuroinflammation, emotional regulation, daytime activity, memory consolidation, and neuroplasticity. Based on a single-center prospective cohort, this study used group-based dual trajectory modeling to identify joint trajectories of sleep quality and cognitive function from pre-discharge to 12 months after discharge, and further examined baseline demographic, clinical, and psychosocial factors associated with trajectory membership. The primary aim was to determine whether AIS survivors could be stratified according to asynchronous sleep–cognition recovery patterns, thereby informing targeted follow-up and individualized post-stroke management.

## 2 Methods

### 2.1 Study design

This study was a single-center prospective cohort study. Based on the longitudinal follow-up data of patients with acute ischemic stroke at T1–T3, GBDTM was used to identify the types of joint evolutionary trajectories of sleep quality and cognitive function (16), and the associations between different joint trajectory subtypes and baseline demographic as well as clinical characteristics were further analyzed.

The analytical framework of this study was based on the central hypothesis that sleep quality and cognitive function after AIS may recover asynchronously as interrelated but non-parallel longitudinal processes. Conventional longitudinal analyses can describe average changes in sleep or cognition separately, but they cannot adequately identify subgroups with discordant patterns, such as improved sleep accompanied by cognitive decline or deteriorated sleep accompanied by cognitive improvement. Therefore, GBDTM was selected because it allows two related repeated-measure outcomes to be modeled simultaneously and identifies latent subgroups defined by their joint developmental patterns. This approach was considered appropriate for addressing the clinical question of whether AIS survivors show heterogeneous sleep–cognition recovery profiles that may require different follow-up strategies.

### 2.2 Study setting and follow-up

This study was conducted at the Tenth People’s Hospital of Shanghai. The enrollment period spanned from December 2023 to October 2024, and consecutive inpatients with acute ischemic stroke were recruited via continuous screening and enrollment. Follow-up and data collection were completed from December 2023 to October 2025. The first assessment (T1) was performed within 24 h before discharge. All participants were followed up for 12 months from the date of discharge, and scale assessments were conducted at 3 months (T2) and 12 months (T3) post-discharge, with a ± 5-day window allowed for each time point; the data cutoff was defined as the time when the last participant completed the 12-month follow-up. All follow-up scale assessments were conducted via researcher home visits; telephone calls were only used for appointment scheduling and travel coordination, but not for cognitive scale evaluation.

### 2.3 Study participants

#### 2.3.1 Inclusion and exclusion criteria

① Aged ≥18 years. ② Diagnosed in accordance with the criteria for acute ischemic stroke after stroke as specified in the Chinese Guidelines for the Diagnosis and Treatment of Acute Ischemic Stroke 2018 (17). ③ *Admitted to ho*spital for treatment within 72 h after acute stroke onset. ④ Stable vital signs and clear consciousness after routine neurological treatment. ⑤ Provided informed consent and voluntarily participated in the study.

#### 2.3.2 Exclusion criteria

① Patients with a history of dementia, mental illness, or intellectual disability. ② Pre-stroke cognitive impairment. ③ Patients who participated in other clinical trials involving unconventional treatments or medications during hospitalization, or those transferred to other hospitals for further treatment. ④ Patients with blindness, deafness, or aphasia. ⑤ Patients residing outside Shanghai.

#### 2.3.3 Exclusion criteria for attrition

① Death of the participant. ② Development of severe complications or other diseases in the participant that prevent the completion of the study. ③ Refusal of the participant to continue participation and request for withdrawal. ④ Loss of contact with the participant. ⑤ Participation of the participant in other clinical trials involving unconventional treatment, medication or rehabilitation after discharge.

### 2.4 Variables, measurements, and data quality

#### 2.4.1 Primary repeated measurement variables

(1) Sleep quality: The Pittsburgh Sleep Quality Index (PSQI) was used to assess sleep quality over the past month, with a total score ranging from 0 to 21; a higher score indicated poorer sleep quality (18). The PSQI assessment was conducted in this study at T1 (24 h before discharge), T2 (3 months post-discharge), and T3 (12 months post-discharge). Referring to the cutoff value commonly used in domestic studies, a total PSQI score > 7 was defined as poor sleep quality (18). To ensure the consistency and comparability of all parameters in the model, a reverse coding process was applied to the PSQI scores for sleep quality evaluation. Specifically, the PSQI was transformed into a form with a positive correlation through the following mathematical formula: Reverse-coded PSQI = Maximum PSQI score (21) − Current PSQI score. This processing ensured that the numerical values of sleep quality were consistent with those of cognitive function in terms of the optimization direction.
(2) Cognitive function: The Montreal Cognitive Assessment (MoCA) was used to evaluate global cognitive function, with a total score ranging from 0 to 30; a higher score indicated better cognitive function (19). MoCA assessments were completed by uniformly trained researchers in a face-to-face manner at T1, T2, and T3, and score corrections were made according to the educational years as recommended by the scale (1 point was added for participants with ≤ 12 years of education).

#### 2.4.2 Covariates

Covariates were mainly collected at T1 for subsequent confounding factor control analysis.

(1) Demographic and sociological information: Age, gender, years of education, occupation, marital status, primary caregiver and other information were obtained by researchers through structured interviews at T1; age and gender were cross-checked with medical record data simultaneously.
(2) Clinical information related to stroke and comorbidities: A history of previous stroke was defined as a clearly diagnosed stroke history before admission, recorded as *yes* or *no*; medical records served as the primary basis, and in case of incomplete medical records, a comprehensive judgment was made by combining statements from patients or their family members with traceable information. Comorbidities such as hypertension, diabetes mellitus and atrial fibrillation were based on clearly diagnosed historical records, and cross-checked with long-term medication history and inpatient medical records.
(3) Disease severity: The National Institutes of Health Stroke Scale (NIHSS) (20) score was extracted from the first standardized assessment within 24 h after admission via the medical record system; a higher score indicated more severe neurological deficit.
(4) Functional status: The Barthel Index (21), Instrumental Activities of Daily Living Scale (IADL) (22), and modified Rankin Scale (mRS) (23) were assessed by uniformly trained researchers at T1 in accordance with the standard procedures of each scale, so as to reflect the pre-discharge functional status. A higher Barthel Index score indicated better basic activities of daily living, whereas a higher mRS score indicated more severe functional impairment. The IADL scale was used to assess the ability to perform more complex activities required for independent community living, including transportation, shopping, meal preparation, medication management, financial management, and household tasks. In the version used in this study, a lower IADL score indicated better instrumental functional status and less dependence, whereas a higher score indicated greater impairment in instrumental activities of daily living.
(5) Lifestyle factors: Information such as smoking and alcohol consumption was obtained through interviews at T1. Both smoking and alcohol consumption were categorized and recorded as never, past and current.
(6) Laboratory indicators: Uric acid (UA), plasma homocysteine (Hcy) and blood lipid indicators (total cholesterol (TC), triglyceride (TG), high-density lipoprotein cholesterol (HDL-C), low-density lipoprotein cholesterol (LDL-C)) were extracted from the electronic inpatient medical record system. Since the follow-up of this study started at discharge and the T1 assessment was completed within 24 h before discharge, laboratory indicators were taken according to a pre-formulated unified rule to align the baseline measurement time point with the follow-up starting point and reduce the time point difference caused by repeated tests during hospitalization: for the same indicator with multiple test results during hospitalization, the last test result within 24 h before discharge was prioritized; if there was no test record within 24 h before discharge, the latest test result during hospitalization closest to the discharge time was selected and the corresponding test time was recorded; the above value-taking rules were uniformly implemented for all participants. Binary variables were constructed based on the above indicators: ① Hyperuricemia: UA ≥ 420 μmol/L in males and UA ≥ 360 μmol/L in females. ② Hyperhomocysteinemia: Hcy > 15 μmol/L. ③ A history of hyperlipidemia: the latest blood lipid diagnostic criteria proposed in the 2012 US Adult Lipid Management Targets were adopted—dyslipidemia indicators included total cholesterol ≥5.2 mmol/L, high-density lipoprotein ≤1.0 mmol/L, triglyceride ≥2.3 mmol/L and low-density lipoprotein ≥2.6 mmol/L; participants with any of the above indicators were diagnosed with hyperlipidemia.
(7) Psychosocial factors: Depressive symptoms were assessed by uniformly trained researchers at T1 through face-to-face interviews using the Chinese version of the Patient Health Questionnaire-9 (PHQ-9) (24), which consisted of 9 items with a total score ranging from 0 to 27; a higher score indicated more severe depressive symptoms. Social support was assessed by face-to-face interviews at T1 using the Social Support Rating Scale (SSRS) (25). The SSRS included 3 dimensions (objective support, subjective support and support utilization) with a total of 10 items and a total score ranging from 12 to 66; a higher score indicated a higher level of social support.

#### 2.4.3 Data sources and quality control

Demographic, lifestyle and psychosocial information were collected by researchers through structured interviews at T1 (24 h before discharge). Clinical data including stroke diagnosis, imaging classification, in-hospital treatment and laboratory examinations were extracted by researchers from the electronic medical record system and uniformly coded according to a preset variable dictionary. The PSQI and MoCA were administered by uniformly trained researchers following standardized procedures at T1, T2 and T3. All follow-up scale assessments were completed via researcher home visits; telephone calls were only used for appointment scheduling and travel coordination, but not for cognitive scale evaluation. Data entry was conducted with double-entry verification and logical checks; random sampling and review were performed for all variables, with a third-party verification implemented when necessary. The preparation and revision of this manuscript were conducted in accordance with the STROBE guidelines, and the STROBE checklist is provided as Supplementary material (26).

#### 2.4.4 Bias control

To reduce selection bias, continuous screening and enrollment were conducted via the medical record system during the enrollment period, and unified inclusion and exclusion criteria were strictly implemented. To mitigate information bias, scales including the PSQI and MoCA were administered by uniformly trained researchers through face-to-face assessments within the specified time window following standardized procedures; variables extracted from medical records were coded uniformly according to a preset dictionary, with double-entry verification and logical checks performed. To assess loss-to-follow-up related bias, the reasons for loss to follow-up were recorded and the loss-to-follow-up rate was reported; where necessary, baseline characteristics were compared between participants lost to follow-up and those who completed the follow-up. Clinically important potential confounding factors were included in the influencing factor analysis for statistical control.

#### 2.4.5 Sample size

This study aimed to identify the joint trajectory subtypes of PSQI and MoCA scores, which was an exploratory analysis. Referring to methodological recommendations for trajectory modeling, to ensure the stability of model estimation and interpretability of trajectories, the minimum proportion of a single trajectory subtype ( $p_{m\text{in}}$ )is generally required to be no less than approximately 5%, and the minimum sample size of a single trajectory subtype ( $n_{m\text{in}}$ ) needs to reach a certain scale to avoid overfitting (13). In this study, $n_{m\text{in}}$ =25 and $\;p_{m\text{in}}$ =0.05. Based on this, the minimum effective sample size ( $N_{m\text{in}}$ )for GBDTM was calculated as follows:


$$N_{m\text{in}}=\frac{n_{m\text{in}}}{p_{m\text{in}}}=\frac{25}{0.05}=500$$

Continuous enrollment was adopted during the study period to meet the above requirement for effective sample size; the final number of participants included in trajectory modeling and the loss-to-follow-up status were reported in the results section.

### 2.5 Statistical methods

To further analyze the relationship between age, marital status, and educational level, we conducted descriptive statistical analysis on the age distribution, marital status, and educational level of the research subjects. We used the χ^2^ Test to examine the correlations between marital status, educational level, and age. This study adopted the GBDTM approach to conduct a dual trajectory analysis of the MoCA scores and reverse-coded PSQI scores, so as to explore the joint evolutionary patterns of cognitive function and sleep quality in the participants (27). Univariate comparative analysis and multivariate Logistic regression were then used to screen the influencing factors of trajectory classification. In addition, the generalized estimating equation (GEE) combined with post-hoc marginal mean comparison was applied to analyze the dynamic changes in major longitudinal outcome indicators among different trajectory groups, so as to provide evidence-based basis for clinical precise intervention. All statistical analyses were performed using SPSS 27.0, and the GBDTM was implemented with R software.

### 2.6 Ethics statement

This study was approved by the Ethics Committee of the Tenth People’s Hospital of Shanghai (Approval No.: SHSY-IEC-5.0/24KY25/P01). Written informed consent was obtained from all participants (or their legal representatives). Study data were de-identified prior to analysis, used solely for research purposes, and securely stored in accordance with institutional requirements. This study was conducted in compliance with the relevant principles of the Declaration of Helsinki (28).

## 3 Results

### 3.1 Age distribution of the research subjects and correlation analysis

**Figure 1:**
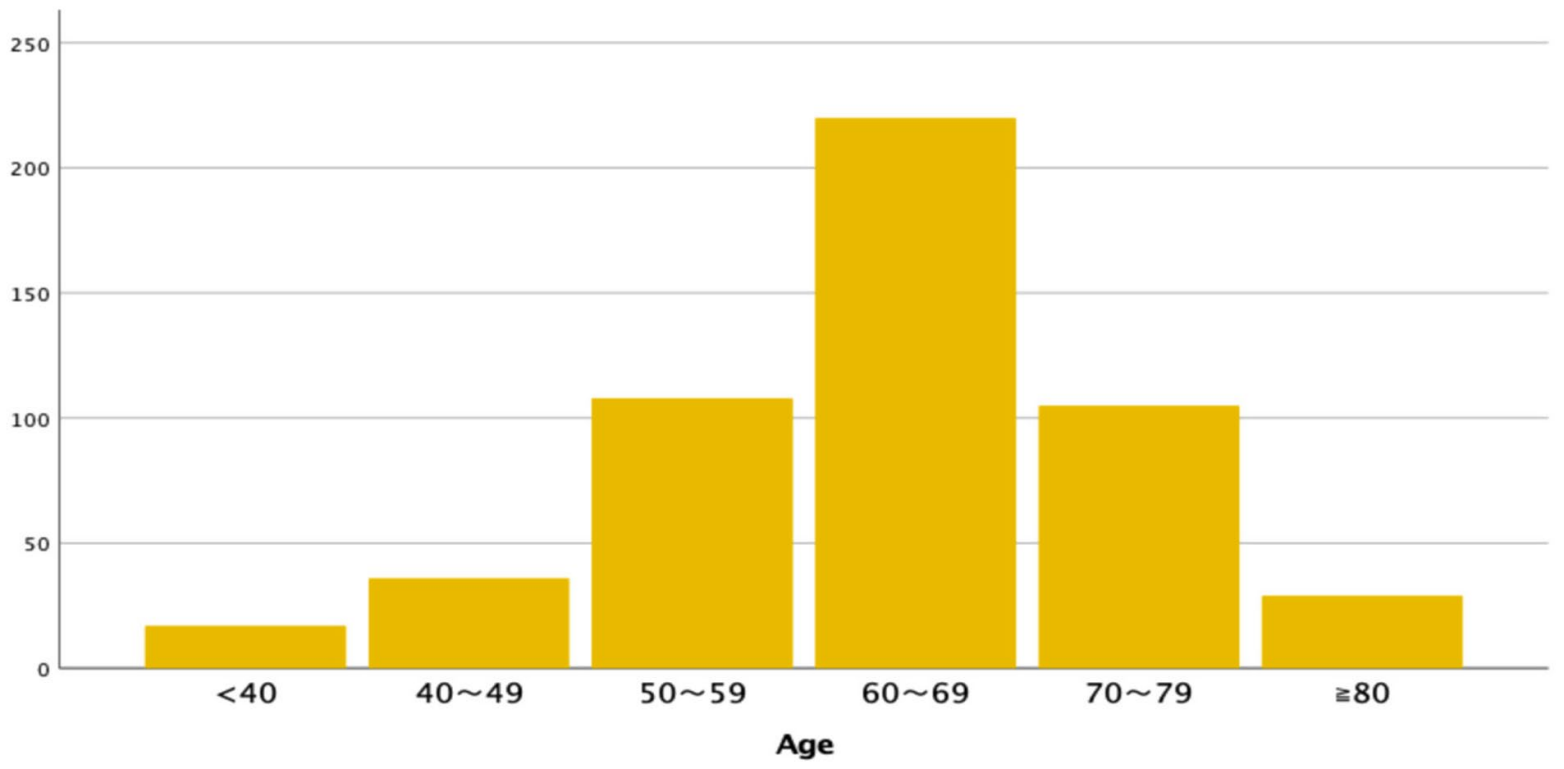
Age distribution of the study population (*N* = 515).

Figure 1 presents the age distribution of the research subjects. The majority of the participants fall within the age group of 60 ~ 69 years, accounting for the largest proportion of the total (*n* = 220, 42.72%), followed by the 50 ~ 59 age group (*n* = 108, 20.97%). The age distribution was right-skewed, with most participants aged 60 years or older.

The results indicated significant associations between age group and both marital status (χ^2^ = 134.755, *p* < 0.001) and educational level (χ^2^ = 93.040, *p* < 0.001). Regarding marital status, unmarried individuals were disproportionately concentrated in the youngest age group (<40 years, 64.71%), whereas this proportion was markedly lower in the 50–59 (5.56%) and 60–69 (9.09%) age groups. Widowed individuals were predominantly found in the ≥80 (24.14%) and 70–79 (9.52%) age groups. Regarding educational level, participants with lower educational attainment (primary school and below, or junior high school) were disproportionately concentrated in the older age groups (70–79 and ≥80 years), while those with higher educational levels (college and above) were more prevalent in younger age groups. These findings confirm that both marital status and educational level are associated with age in this cohort; accordingly, both variables were retained as covariates in the subsequent multivariate logistic regression model to control for potential confounding. The detailed age-stratified distribution of marital status and educational level, as shown in Table 1, provides additional insights.

**Table 1:** The age distribution of the research population and its correlation with marital status and educational level (n, %).

Indicator	<40 year	40 ~ 49 year	50 ~ 59 year	60 ~ 69 year	70 ~ 79 year	≧80 year	χ^2^	*P*
Marital status							134.755	<0.001***
Married	6(35.29%)	36 (100.00%)	99 (91.67%)	196 (89.09%)	95 (90.48%)	22 (75.86%)		
Unmarried	11 (64.71%)	0 (0.00%)	6 (5.56%)	20 (9.09%)	0 (0.00%)	0 (0.00%)		
Widowed	0 (0.00%)	0 (0.00%)	3 (2.78%)	4 (1.82%)	10 (9.52%)	7 (24.14%)		
Education level							93.040	<0.001***
Primary school and below	0 (0.00%)	0 (0.00%)	12 (11.11%)	13 (5.91%)	9 (8.57%)	2 (6.90%)		
Junior high school	0 (0.00%)	8 (22.22%)	27 (25.00%)	98 (44.55%)	38 (36.19%)	6 (20.69%)		
Senior high school	3 (17.65%)	9 (25.00%)	54 (50.00%)	73 (33.18%)	32 (30.48%)	18 (62.07%)		
College and above	14 (82.35%)	19 (52.78%)	15 (13.89%)	36 (16.36%)	26 (24.76%)	3 (10.34%)		

***indicates a *p*-value < 0.001.

### 3.2 The changes in the MoCA and PSQI scores of the participants

To assess the changes in cognitive function and sleep quality, we analyzed the MoCA and PSQI scores of the participants at baseline (T1), 3 months (T2), and 12 months (T3). Table 2 presents the mean and standard deviation of the MoCA and PSQI scores at each time point, showing that both scores increased over time. As shown in Table 2, both MoCA and PSQI scores increased over time. The MoCA score at T1 was 16.73 ± 5.27, and increased slightly to 17.56 ± 5.81 at T3, suggesting a modest improvement in cognitive function. Similarly, the PSQI score increased from 14.78 ± 3.76 at T1 to 16.30 ± 3.18 at T3, indicating a slight deterioration in sleep quality over the 12-month period. These changes, although modest, reflect the participants’ overall cognitive and sleep status.

**Table 2:** The changes in the MoCA and PSQI scores of the participants.

Time	PSQI	MoCA
T1	14.78 ± 3.76	16.73 ± 5.27
T2	15.59 ± 3.03	16.64 ± 6.14
T3	16.30 ± 3.18	17.56 ± 5.81

### 3.3 Dual trajectory model fitting and determination of the optimal number of classes

The model fit statistics for the one- to five-class dual trajectory models are summarized in Table 3. Results of the dual trajectory analysis showed that models with different class numbers all achieved convergence. The optimal number of trajectory classes was comprehensively determined based on information criteria (AIC, BIC, CAIC, ssBIC, hqic), classification consistency coefficient (appa) and entropy value (ek). Although the one-class model had an appa value of 1.000, it presented the highest information criterion value (AIC = 7108.854) and failed to reflect population heterogeneity. The information criterion values of the 2–5-class models gradually decreased with the increase of class numbers. Among them, the five-class model had the lowest AIC (5137.305), but its entropy value (0.9260) was lower than that of the four-class model (0.9318); in addition, it showed scattered class proportion distribution (13.20–33.20%) with poor clinical interpretability. The three-class model had a higher AIC (6145.139) than the four and five-class models, yet it reached an entropy value of 0.8990 with good classification consistency (appa values were 0.941, 0.986 and 0.932 for each class respectively). It also presented a balanced and reasonable class proportion distribution: 152 cases (29.51%), 223 cases (43.30%) and 140 cases (27.18%) for each class, with clear clinical implications. Therefore, the three-class trajectory was finally identified as the optimal classification scheme.

**Table 3:** Estimation results of the dual trajectory model.

Number of classes	Polynomial order	aic	bic	caic	ssbic	hqic	appa	ek	occ	Class proportion
1	1	7,108.854	7,146.253	7,153.253	7,124.016	7,122.766	1.000		–	515 (100%)
2	1	6,528.044	6,602.843	6,616.843	6,558.368	6,555.868	0.969/0.924	0.8455	18.530/20.786	320 (62.14%)/195 (37.86%)
3	1	6,145.139	6,268.023	6,291.023	6,194.958	6,190.850	0.941/0.986/0.932	0.8990	39.716/88.750/37.980	152 (29.51%)/223 (43.30%)/140 (27.18%)
4	1	5,488.242	5,653.868	5,684.868	5,555.389	5,549.852	0.992/0.991/0.967/0.885	0.9318	160.756/534.318/128.182/31.457	217 (42.14%)/87 (16.89%)/99 (19.22%)/112 (21.75%)
5	1	5,137.305	5,345.674	5,384.674	5,221.780	5,214.815	0.976/0.990/0.951/0.988/0.898	0.9260	75.865/501.930/120.488/558.033/33.204	171 (33.20%)/87 (16.89%)/73 (14.17%)/68 (13.20%)/116 (22.52%)

**Figure 2:**
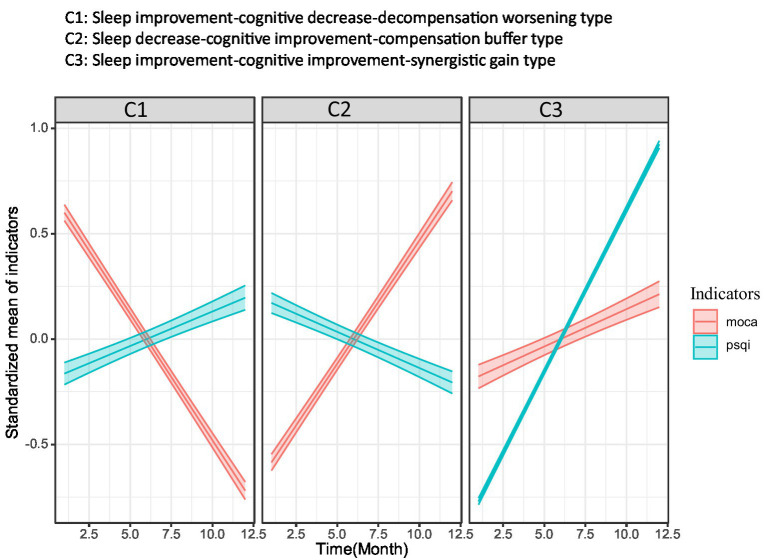
Estimated class-specific dual trajectories of MoCA and PSQI from the joint trajectory model.

The class-specific dual trajectories of MoCA and PSQI are illustrated in Figure 2. Trajectory visualization revealed significant differences in the temporal evolution trends of MoCA and PSQI scores among the three classes, reflecting distinct joint development patterns of cognitive function and sleep quality. The three classes of the dual trajectory model were named according to their characteristic score changes: Class 1 (C1) was defined as the sleep improvement-cognitive decline-decompensation deterioration type, in which patients showed an increasing trend in sleep scores but a decreasing trend in cognitive scores; Class 2 (C2) was the sleep decline-cognitive improvement-compensation buffering type, with patients presenting a decreasing sleep score trend and an increasing cognitive score trend; Class 3 (C3) was the sleep improvement-cognitive improvement-synergistic gain type, where patients exhibited an increasing trend in both sleep quality and cognitive scores. In Figure 2, the shaded areas represent the standard deviation (SD) of the estimated trajectories, indicating the variability around the mean trajectories for each class. These areas reflect the uncertainty of the trajectory estimates at each time point.

### 3.4 Univariate comparison of clinical characteristics among different trajectory groups

Chi-square test, Fisher’s exact probability test and one-way analysis of variance (ANOVA) were used to compare the general data of patients among different groups, and the results showed statistically significant differences in some indicators (*p* < 0.05). As shown in Table 4, the detailed intergroup comparisons of demographic and clinical characteristics reveal statistically significant differences in some indicators.

**Table 4:** Intergroup comparison of general data and clinical indicators.

Indicator	C1(*n* = 152)	C2(*n* = 223)	C3 (*n* = 140)	*χ^2^/F/Z*	*p*-value
Age [M (Q1, Q3)]	64.00 (59.00,69.00)	64.00 (57.00,71.00)	64.00 (58.25,69.75)	0.505	0.777
Gender [*n* (%)]				1.031	0.597
Female	82 (53.95%)	132 (59.19%)	79 (56.43%)		
Male	70 (46.05%)	91 (40.81%)	61 (43.57%)		
Marital status [*n* (%)]				15.265	0.004**
Married	136 (89.47%)	205 (91.93%)	113 (80.71%)		
Unmarried	6 (3.95%)	13 (5.83%)	18 (12.86%)		
Widowed	10 (6.58%)	5 (2.24%)	9 (6.43%)		
Education level [*n* (%)]				5.564	0.474
Primary school and below	16 (10.53%)	12 (5.38%)	8 (5.71%)		
Junior high school	51 (33.55%)	79 (35.43%)	47 (33.57%)		
Senior high school	55 (36.18%)	78 (34.98%)	56 (40.00%)		
College and above	30 (19.74%)	54 (24.22%)	29 (20.71%)		
Occupation [*n* (%)]				1.053	0.591
Retired	121 (79.61%)	175 (78.48%)	116 (82.86%)		
Employed	31 (20.39%)	48 (21.52%)	24 (17.14%)		
Caregiver relationship [*n* (%)]				12.499	0.130
Spouse	57 (37.50%)	86 (38.57%)	42 (30.00%)		
Children	18 (11.84%)	27 (12.11%)	24 (17.14%)		
Spouse or children	58 (38.16%)	69 (30.94%)	44 (31.43%)		
Other relatives	9 (5.92%)	30 (13.45%)	18 (12.86%)		
Nursing worker	10 (6.58%)	11 (4.93%)	12 (8.57%)		
Medical insurance [*n* (%)]				10.888	0.004**
Self-paid	127 (83.55%)	205 (91.93%)	132 (94.29%)		
Medical insurance	25 (16.45%)	18 (8.07%)	8 (5.71%)		
Thrombolysis [*n* (%)]				5.557	0.062
Yes	130 (85.53%)	174 (78.03%)	121 (86.43%)		
No	22 (14.47%)	49 (21.97%)	19 (13.57%)		
Infarction location [*n* (%)]				27.497	0.002**
Vertebrobasilar artery	36 (23.68%)	76 (34.08%)	35 (25.00%)		
Bilateral cerebral hemispheres	11 (7.24%)	13 (5.83%)	13 (9.29%)		
Right cerebral hemisphere	18 (11.84%)	45 (20.18%)	30 (21.43%)		
Left cerebral hemisphere	47 (30.92%)	39 (17.49%)	22 (15.71%)		
Watershed area	13 (8.55%)	6 (2.69%)	10 (7.14%)		
Other locations	27 (17.76%)	44 (19.73%)	30 (21.43%)		
Corrected MoCA [*n* (%)]	54 (35.53%)	93 (41.70%)	44 (31.43%)	4.117	0.128
Sedentary lifestyle [*n* (%)]	148 (97.37%)	218 (97.76%)	139 (99.29%)		0.509
Smoking status [*n* (%)]	78 (51.32%)	99 (44.39%)	71 (50.71%)	2.239	0.327
Alcohol consumption [*n* (%)]	58 (38.16%)	71 (31.84%)	47 (33.57%)	1.636	0.441
Internal carotid artery stenosis [*n* (%)]	24 (15.79%)	38 (17.04%)	28 (20.00%)	0.948	0.623
Intracranial arterial stenosis [*n* (%)]	28 (18.42%)	35 (15.70%)	37 (26.43%)	6.469	0.039*
Carotid arteriosclerosis [*n* (%)]	32 (21.05%)	40 (17.94%)	36 (25.71%)	3.140	0.208
Arrhythmia [*n* (%)]	7 (4.61%)	27 (12.11%)	14 (10.00%)	6.125	0.047*
Hyperuricemia [*n* (%)]	15 (9.87%)	11 (4.93%)	14 (10.00%)	4.412	0.110
Hypertension [*n* (%)]	108 (71.05%)	145 (65.02%)	89 (63.57%)	2.167	0.338
Hyperhomocysteinemia [*n* (%)]	38 (25.00%)	59 (26.46%)	38 (27.14%)	0.185	0.912
Diabetes mellitus [*n* (%)]	60 (39.47%)	89 (39.91%)	60 (42.86%)	0.420	0.811
Coronary heart disease [*n* (%)]	22 (14.47%)	30 (13.45%)	18 (12.86%)	0.169	0.919
Atrial fibrillation [*n* (%)]	4 (2.63%)	31 (13.90%)	9 (6.43%)	15.793	<0.001***
Hyperlipidemia [*n* (%)]	29 (19.08%)	53 (23.77%)	37 (26.43%)	2.312	0.315
TIA [*n* (%)]	7 (4.61%)	10 (4.48%)	7 (5.00%)	0.053	0.974
History of stroke [*n* (%)]	27 (17.76%)	18 (8.07%)	20 (14.29%)	8.181	0.017*
Thyroid disease [*n* (%)]	5 (3.29%)	17 (7.62%)	7 (5.00%)	3.339	0.188

*indicates a *P*-value < 0.05; **indicates a *P*-value < 0.01; ***indicates a *P*-value < 0.001.

Among demographic characteristics, marital status (χ^2^ = 15.265, *p* = 0.004) and medical insurance type (χ^2^ = 10.888, *p* = 0.004) presented significant intergroup differences: the C2 trajectory group had the highest proportion of married patients (91.93%), while the C3 trajectory group had the highest proportion of unmarried patients (12.86%); the C3 trajectory group had the highest proportion of self-funded medical insurance (94.29%), and the C1 trajectory group had the highest proportion of social medical insurance (16.45%). No statistically significant differences were found in gender, educational level, occupation and caregiver type among the groups (all *p* > 0.05).

Among clinical disease indicators, significant intergroup differences were observed in infarction location (χ^2^ = 27.497, *p* = 0.002), intracranial arterial stenosis (χ^2^ = 6.469, *p* = 0.039), arrhythmia (χ^2^ = 6.125, *p* = 0.047), atrial fibrillation (χ^2^ = 15.793, *p* < 0.001) and **history of stroke** (χ^2^ = 8.181, *p* = 0.017). The C2 trajectory group had the highest proportion of vertebrobasilar artery infarction (34.08%), the highest incidence of atrial fibrillation (13.90%) and the lowest proportion of stroke history (8.07%); the C3 trajectory group had the highest proportion of intracranial arterial stenosis (26.43%); the C1 trajectory group had the highest proportion of left cerebral hemisphere infarction (30.92%). No statistically significant intergroup differences were found in thrombolytic therapy and other comorbidities including hypertension, diabetes mellitus, coronary heart disease and hyperlipidemia (all *p* > 0.05), suggesting that the above indicators with significant differences may be associated with the classification of cognitive-sleep dual trajectories.

### 3.5 Multivariate logistic regression analysis of influencing factors for trajectory classification

C1 was selected as the reference group in the multinomial logistic regression model because it represented a clinically important discordant trajectory characterized by improvement in sleep quality but decline in cognitive function. This pattern suggests that cognitive deterioration may occur despite apparent improvement in sleep quality, and therefore provides a meaningful comparator for identifying factors associated with alternative joint sleep–cognition trajectories. Accordingly, the odds ratios for C2 and C3 should be interpreted as the relative likelihood of belonging to the sleep decline–cognitive improvement trajectory or the sleep improvement–cognitive improvement trajectory, respectively, compared with the C1 sleep improvement–cognitive decline trajectory. For the C2 trajectory group (compared with C1), widowhood, left cerebral hemisphere infarction and history of stroke were identified as associated with lower odds of class membership, while arrhythmia, atrial fibrillation and PSQI_T1 were associated with higher odds of class membership. For the C3 trajectory group compared with C1, unmarried status was associated with higher odds of C3 membership, whereas left cerebral hemisphere infarction, watershed infarction, social medical insurance, and PSQI_T1 were associated with lower odds of C3 membership (all *p* < 0.05). The detailed odds ratios, 95% confidence intervals, and *p*-values for the multinomial logistic regression model are shown in Table 5.

**Table 5:** Multivariate logistic regression model for predicting trajectory classification.

Outcome	Indicator	OR	95% CI	*p*-value
C2	Marital status			
	Married	–	–	
	Unmarried	1.34	0.43, 4.19	0.619
	Widowed	0.29	0.09, 0.98	0.047*
	Medical insurance			
	Self-funded	–	–	
	Insured	0.60	0.28, 1.30	0.199
	Infarction location			
	Vertebrobasilar artery	–	–	
	Bilateral cerebral hemispheres	0.53	0.19, 1.44	0.212
	Right cerebral hemisphere	1.39	0.67, 2.90	0.378
	Left cerebral hemisphere	0.49	0.25, 0.95	0.036*
	Watershed area	0.40	0.13, 1.26	0.118
	Other locations	0.57	0.28, 1.17	0.127
	Intracranial arterial stenosis			
	No	–	–	
	Yes	0.79	0.42, 1.50	0.475
	Arrhythmia			
	No	–	–	
	Yes	4.15	1.60, 10.8	0.003**
	Atrial fibrillation			
	No	–	–	
	Yes	9.85	3.17, 30.7	<0.001***
	History of stroke			
	No	–	–	
	Yes	0.28	0.13, 0.60	0.001***
	Baseline social support	0.98	0.95, 1.01	0.121
	PSQI_T1	1.21	1.12, 1.30	<0.001***
C3	Marital status			
	Married	–	–	
	Unmarried	6.64	1.99, 22.2	0.002**
	Widowed	0.88	0.27, 2.85	0.837
	Medical insurance			
	Self-funded	–	–	
	Insured	0.32	0.12, 0.88	0.027*
	Infarction location			
	Vertebrobasilar artery	–	–	
	Bilateral cerebral hemispheres	0.95	0.31, 2.86	0.925
	Right cerebral hemisphere	1.43	0.61, 3.36	0.406
	Left cerebral hemisphere	0.33	0.14, 0.77	0.010**
	Watershed area	0.29	0.09, 0.96	0.043*
	Other locations	0.78	0.33, 1.82	0.565
	Intracranial arterial stenosis			
	No	–	–	
	Yes	0.84	0.42, 1.68	0.615
	Arrhythmia			
	No	–	–	
	Yes	0.92	0.29, 2.89	0.886
	Atrial fibrillation			
	No	–	–	
	Yes	2.59	0.70, 9.60	0.155
	History of stroke			
	No	–	–	
	Yes	0.63	0.28, 1.44	0.273
	Baseline social support	0.98	0.95, 1.02	0.332
	PSQI_T1	0.77	0.70, 0.84	<0.001***

PSQI_T1, Assessment of sleep quality during hospitalization. *indicates a *P*-value < 0.05; **indicates a *P*-value < 0.01; ***indicates a *P*-value < 0.001.

### 3.6 GEE model analysis and marginal mean comparison of major longitudinal outcome indicators

A GEE model was applied to analyze the dynamic changes in four core longitudinal indicators, namely the National Institutes of Health Stroke Scale (NIHSS), the Barthel Index, instrumental activities of daily living (IADL) scores, and Patient Health Questionnaire-9 (PHQ-9) scores, across different trajectory groups. Time was set as the longitudinal variable, posterior classification as the grouping variable, and the time × posterior classification interaction term was included to explore intergroup differences in the changing trends of the indicators. Post-hoc marginal mean comparisons were further performed to verify intergroup differences at different time points.

**Figure 3:**
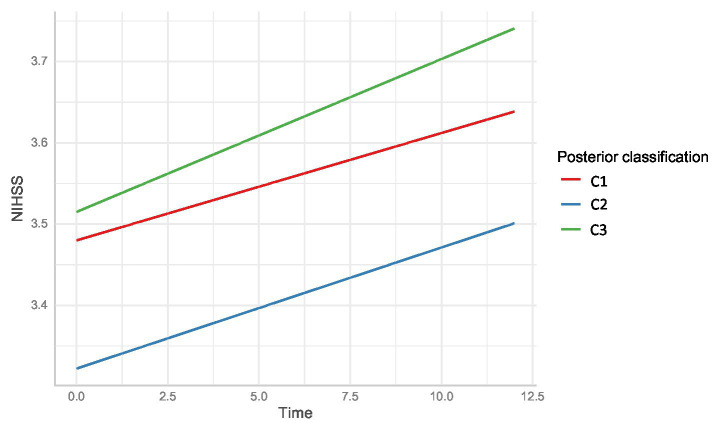
Longitudinal changes in NIHSS over time by posterior trajectory class.

NIHSS scores showed no significant longitudinal changes across the three trajectory groups (Figure 3). The GEE analysis revealed no significant main effect of posterior trajectory classification and no significant time × posterior classification interaction (Table 6). Post-hoc marginal mean comparisons further confirmed no significant between-class differences at any follow-up time point (Table 7).

**Table 6:** Results of the generalized estimating equation model for NIHSS.

Indicator estimate	Indicator estimate	95% CI	*p*-value
Time	0.01	−0.005, 0.032	0.166
Posterior classification			
C1	–	–	
C2	−0.16	−0.445, 0.129	0.281
C3	0.04	−0.276, 0.346	0.825
Time * posterior classification			
Time * C2	0.00	−0.022, 0.025	0.888
Time * C3	0.01	−0.022, 0.034	0.694

CI, confidence interval.

**Table 7:** Intergroup comparison of *post hoc* marginal means for NIHSS.

Time	Comparison groups	Difference estimate	Standard error	Test statistic	*p*-value
T0	C2–C1	−0.158	0.146	−1.078	0.281
T0	C3–C1	0.035	0.159	0.221	0.825
T0	C3–C2	0.193	0.136	1.421	0.155
T1	C2–C1	−0.153	0.172	−0.887	0.375
T1	C3–C1	0.052	0.180	0.288	0.773
T1	C3–C2	0.205	0.158	1.293	0.196
T2	C2–C1	−0.137	0.264	−0.520	0.603
T2	C3–C1	0.102	0.275	0.372	0.710
T2	C3–C2	0.240	0.250	0.958	0.338

**Figure 4:**
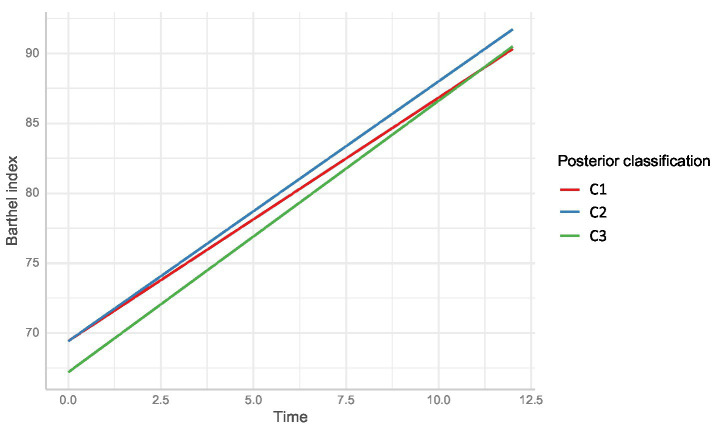
Longitudinal changes in the Barthel Index over time by posterior trajectory class.

Barthel Index scores increased over time, indicating improvement in basic activities of daily living (Figure 4). The GEE model showed a significant main effect of time (Table 8). However, there were no significant between-class differences or time × posterior classification interactions (Table 8), suggesting that basic functional recovery followed a similar pattern across the three trajectory groups. Post-hoc comparisons further confirmed the absence of significant differences at any time point (Table 9).

**Table 8:** Results of the generalized estimating equation model for Barthel Index.

Indicator	Estimate	95% CI	*p*-value
Time	1.7	1.552, 1.928	<0.001***
Posterior classification			
C1	–	–	
C2	−0.02	−3.624, 3.590	0.993
C3	−2.2	−6.176, 1.705	0.266
Time * posterior classification			
Time * C2	0.12	−0.122, 0.360	0.334
Time * C3	0.20	−0.052, 0.456	0.120

***indicates a *P*-value < 0.001.

**Table 9:** Intergroup comparison of *post hoc* marginal means for Barthel Index.

Time	Comparison groups	Difference estimate	Standard error	Test statistic	*p*-value
T0	C2–C1	−0.017	1.840	−0.009	0.993
T0	C3–C1	−2.236	2.011	−1.112	0.266
T0	C3–C2	−2.219	1.740	−1.275	0.202
T1	C2–C1	0.339	1.675	0.203	0.839
T1	C3–C1	−1.630	1.851	−0.881	0.379
T1	C3–C2	−1.970	1.555	−1.267	0.205
T2	C2–C1	1.408	1.641	0.858	0.391
T2	C3–C1	0.186	1.843	0.101	0.920
T2	C3–C2	−1.223	1.429	−0.856	0.392

**Figure 5:**
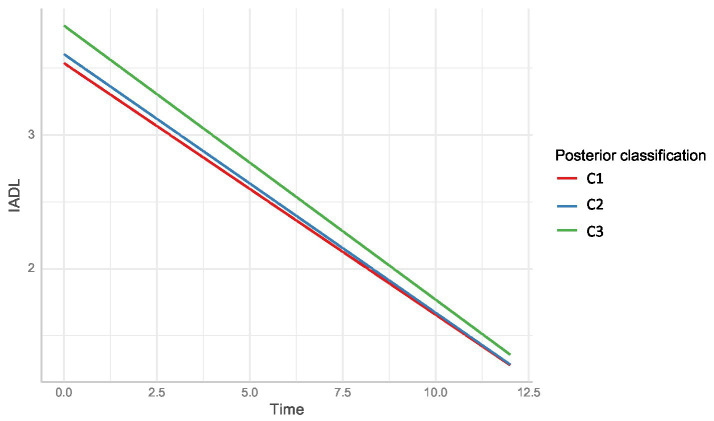
Longitudinal changes in IADL scores over time by posterior trajectory class.

IADL scores changed over time (Figure 5). The GEE model showed a significant main effect of time (Table 10), indicating longitudinal changes in instrumental activities of daily living during follow-up. However, neither the main effect of posterior trajectory classification nor the time × posterior classification interaction was statistically significant (Table 10). Post-hoc marginal mean comparisons confirmed that IADL changes occurred similarly across the three joint sleep–cognition trajectory groups (Table 11). Because a lower IADL score indicated better instrumental functional status in the version used in this study, the significant negative time effect for IADL should be interpreted as an improvement in instrumental activities of daily living over the follow-up period.

**Table 10:** Results of the generalized estimating equation model for IADL.

Indicator	Estimate	95% CI	*p*-value
Time	−0.19	−0.213, −0.163	<0.001***
Posterior classification			
C1	–	–	
C2	0.07	−0.383, 0.516	0.772
C3	0.28	−0.199, 0.760	0.252
Time * posterior classification			
Time * C2	−0.01	−0.038, 0.027	0.752
Time * C3	−0.02	−0.053, 0.019	0.359

***indicates a *P*-value < 0.001.

**Table 11:** Intergroup comparison of *post hoc* marginal means for IADL.

Time	Comparison groups	Difference estimate	Standard error	Test statistic	*p*-value
T0	C2–C1	0.067	0.229	0.290	0.772
T0	C3–C1	0.281	0.245	1.147	0.252
T0	C3–C2	0.214	0.220	0.972	0.331
T1	C2–C1	0.051	0.201	0.253	0.800
T1	C3–C1	0.230	0.215	1.069	0.285
T1	C3–C2	0.179	0.190	0.942	0.346
T2	C2–C1	0.004	0.182	0.019	0.985
T2	C3–C1	0.078	0.205	0.382	0.702
T2	C3–C2	0.075	0.177	0.422	0.673

**Figure 6:**
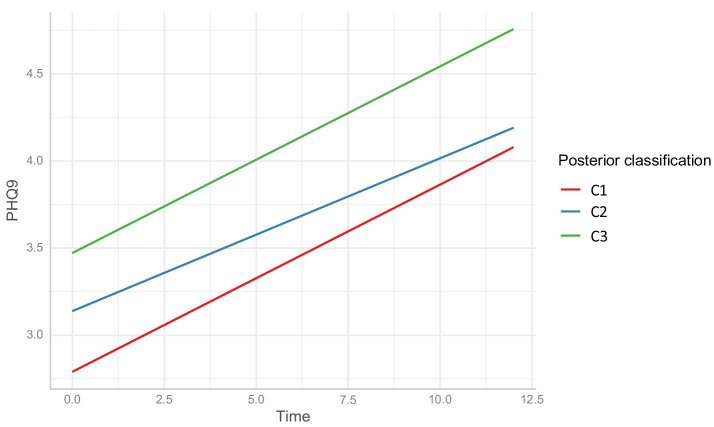
Longitudinal changes in PHQ-9 scores over time by posterior trajectory class.

PHQ-9 scores increased over time across the follow-up period (Figure 6). The GEE analysis demonstrated a significant main effect of time (Table 12) (estimate = 0.11, *p* < 0.001). Post-hoc marginal mean comparisons showed significantly higher PHQ-9 scores in the C3 group than in the C1 group at T1 and T2 (Table 13) (*p* = 0.023 and *p* = 0.021, respectively).

**Table 12:** Results of the generalized estimating equation model for PHQ-9.

Indicator	Estimate	95% CI	*p*-value
Time	0.11	0.074, 0.141	<0.001***
Posterior classification			
C1	–	–	
C2	0.35	−0.197, 0.895	0.210
C3	0.68	0.095, 1.269	0.023*
Time * posterior classification			
Time * C2	−0.02	−0.066, 0.026	0.401
Time * C3	0.00	−0.051, 0.050	0.990

* indicates a *P*-value < 0.05, and *** indicates a *P*-value < 0.001.

**Table 13:** Intergroup comparison of marginal means for PHQ-9.

Time	Comparison groups	Difference estimate	Standard error	Test statistic	*p*-value
T0	C2–C1	0.349	0.279	1.252	0.210
T0	C3–C1	0.682	0.299	2.278	0.023*
T0	C3–C2	0.333	0.294	1.134	0.257
T1	C2–C1	0.290	0.276	1.051	0.293
T1	C3–C1	0.681	0.294	2.314	0.021*
T1	C3–C2	0.391	0.272	1.439	0.150
T2	C2–C1	0.112	0.361	0.310	0.756
T2	C3–C1	0.678	0.387	1.751	0.080
T2	C3–C2	0.566	0.328	1.727	0.084

* indicates a *P*-value < 0.05.

## 4 Discussion

### 4.1 Key findings and clinical implications of joint trajectories: asynchronous recovery of sleep and cognition after stroke

The key clinical message of this study is that recovery after AIS should not be inferred from a single domain. Using group-based dual trajectory modeling, we identified three clinically interpretable sleep–cognition recovery patterns during the first year after discharge: sleep improvement with cognitive decline, sleep decline with cognitive improvement, and simultaneous sleep and cognitive improvement. These findings indicate that improvement in one recovery domain does not necessarily represent global recovery. In clinical follow-up, a patient with apparently improved sleep may still experience cognitive deterioration, whereas a patient with improving cognition may still have worsening sleep quality. Therefore, sleep quality and cognitive function should be assessed in parallel rather than treated as secondary, interchangeable, or automatically synchronized outcomes. This interpretation is clinically important because routine post-stroke follow-up often prioritizes neurological impairment, basic activities of daily living, and recurrence prevention, while sleep and cognition may be evaluated separately or only when patients actively report symptoms. The present findings suggest that such a single-domain approach may miss patients with discordant recovery profiles who require different follow-up priorities.

The three-class solution was also clinically useful because it did not merely divide patients into statistically distinct subgroups; it generated follow-up categories with different practical implications. The sleep improvement–cognitive decline group represents patients whose cognitive deterioration may be hidden if follow-up focuses only on sleep complaints or general functional recovery. The sleep decline–cognitive improvement group represents patients whose cognitive recovery may coexist with unresolved or worsening sleep problems. The sleep improvement–cognitive improvement group represents a relatively favorable recovery pattern, but this group still requires emotional monitoring because PHQ-9 scores were higher in C3 than in C1 at early follow-up. Thus, the value of the three-class model lies in its clinical translatability: it helps convert heterogeneous longitudinal changes into distinct follow-up priorities rather than simply describing statistical heterogeneity.

The observed discordant trajectories are clinically plausible because sleep and cognition are related but not identical recovery domains. Sleep disturbance after stroke may be influenced by brain injury, hospitalization, pain, medication effects, emotional distress, reduced daytime activity, nocturia, and possible sleep-disordered breathing. At the same time, poor sleep may impair attention, executive function, learning efficiency, memory consolidation, and neural network remodeling through circadian rhythm disruption, inflammatory activation, autonomic dysregulation, and stress-axis activation. However, the present results show that these mechanisms do not always produce parallel clinical change. Some patients may achieve cognitive improvement despite worsening sleep, possibly because cognitive recovery is supported by rehabilitation, spontaneous neurological recovery, or cognitive reserve, while sleep remains affected by behavioral, emotional, or environmental factors. Conversely, some patients may report better sleep while cognition declines, suggesting that subjective sleep improvement should not be used as a proxy for cognitive safety. The clinical interpretation is therefore straightforward: sleep and cognition should be monitored as two interacting but partially independent targets of post-stroke recovery.

### 4.2 Baseline factors as practical markers for stratified sleep–cognition follow-up

The baseline factors associated with trajectory membership should be interpreted primarily as practical markers for stratified follow-up rather than as direct causal determinants of sleep–cognition recovery. In this study, marital status, medical insurance type, infarction location, arrhythmia, atrial fibrillation, history of stroke, and baseline sleep quality helped characterize patients who were more likely to enter different joint trajectories. Clinically, these variables may help identify patients who require more intensive sleep assessment, cognitive surveillance, emotional screening, caregiver evaluation, or cardiovascular risk management after discharge. This interpretation is more useful than treating each variable as an isolated risk factor, because the main clinical value of the model is to support differentiated follow-up priorities according to the patient’s recovery pattern.

Marital status was associated with trajectory affiliation, but this finding should be interpreted cautiously because marital status was closely related to age structure and life stage in this cohort. The additional age-stratified analysis showed that unmarried status was disproportionately represented in the younger age strata, while widowed status was more common in older age groups. Consistently, the C3 trajectory group, characterized by simultaneous improvement in sleep quality and cognitive function, had the highest proportion of unmarried participants. Therefore, the higher likelihood of C3 membership among unmarried patients should not be interpreted as evidence that unmarried status itself promotes coordinated sleep–cognitive improvement. A more plausible interpretation is that unmarried status in this cohort partly reflected a younger life-stage profile, which may be accompanied by better premorbid functional reserve, fewer age-related cognitive vulnerabilities, greater independence in daily routines, and different post-discharge activity and sleep patterns. This point is important because the primary focus of the present study was not to establish causal effects of social-demographic characteristics, but to identify heterogeneous joint sleep–cognition trajectories and describe baseline factors associated with trajectory membership. Accordingly, marital status should be regarded as a contextual marker rather than a direct intervention target. The clinical implication is not to stratify patients simply by whether they are married or unmarried, but to assess the underlying modifiable conditions that marital status may proxy, including actual caregiver availability, living arrangement, rehabilitation adherence, daytime activity structure, emotional support, and follow-up accessibility. In this sense, the finding concerning unmarried patients in the C3 group helps characterize the demographic context of the co-improvement trajectory, while also reinforcing the need to avoid overinterpreting social-demographic variables as independent causal mechanisms.

Medical insurance type was associated with trajectory affiliation, indicating the critical role of economic security in long-term rehabilitation. This is consistent with the conclusions of the study by Larson et al. (29): socioeconomic status and medical insurance often influence long-term outcomes through three pathways of “accessibility-persistence-intensity.” Follow-up visits, rehabilitation training, and sleep and emotion-related interventions after stroke all require sustained investment; economic pressure not only limits access to medical services, but also indirectly affects cognitive recovery through anxiety, depression and sleep disorders. A previous study by O’Callaghan et al. (30) also identified a practical paradox: functional indicators appear to improve during hospitalization, but some patients experience persistent deterioration or recurrence in sleep, emotion and cognition after discharge due to the combined costs of follow-up visits, rehabilitation and caregiving. The results of the present study showed a significant intergroup difference in medical insurance coverage across trajectory groups, and being covered by medical insurance was associated with more favorable trajectory membership. This has specific implications for nursing management: for individuals with inadequate economic security, a one-size-fits-all approach of “standard follow-up” should not be adopted; instead, sustained support should be provided through lower-threshold methods (telephone/online follow-up, reproducible behavioral prescriptions, family care guidance), otherwise they are more likely to enter an adverse trajectory in the early post-discharge period. Furthermore, several previous intervention studies have suggested that low-cost, implementable and supervisable programs (e.g., behavioral prescriptions plus follow-up supervision) are more likely to produce real effects in resource-constrained populations, which also supports the stratified follow-up strategy proposed in this study.

The association between arrhythmia/atrial fibrillation and trajectory affiliation highlights the importance of the heart-brain-sleep interaction. A previous study by Huang et al. (31) has clearly indicated that atrial fibrillation is associated with post-stroke cognitive impairment and an increased risk of cognitive decline, which may be attributed to cardiogenic embolism, fluctuating cerebral perfusion, occult microembolic load, and an underlying background of cerebral small vessel disease. Meanwhile, the experience of cardiovascular symptoms, medication complexity and nocturnal discomfort may also directly disrupt sleep maintenance. Notably, previous studies by Jiang et al. and Bottignole et al. (3, 32) have also suggested a strong comorbidity between cardiovascular diseases and sleep-disordered breathing; sleep-disordered breathing events can exacerbate autonomic dysfunction, impair nocturnal oxygenation and cerebral blood flow regulation, thereby forming a negative feedback loop in terms of sleep and cognition. Based on this, sleep problems should be incorporated into the overall risk assessment for stroke patients with atrial fibrillation/arrhythmia. Nursing follow-up needs to focus on clues such as persistent poor sleep, daytime sleepiness and nocturnal awakening due to dyspnea, and facilitate further evaluation and referral when necessary. The key here is “pathway-based management”: integrating cardiac rhythm risk management with sleep screening, emotional assessment and cognitive monitoring into the same follow-up closed loop, rather than each specialty being responsible for a separate segment of care.

It is even more noteworthy that functional recovery after stroke should be interpreted at different levels. Basic functional scales, such as the Barthel Index, mainly reflect self-care abilities, including feeding, dressing, toileting, transferring, and mobility. These functions may improve significantly over time; however, such improvement does not necessarily indicate coordinated recovery of sleep, cognition, mood, or higher-order functional capacity. Consistent with previous stroke follow-up studies, basic physical recovery tends to occur earlier and more visibly, whereas sleep, cognition, mood, and instrumental functional capacity are more latent dimensions that are easily overlooked and may be influenced by lifestyle, family structure, psychological stress, and environmental demands (13, 15, 33, 34).

The interpretation of IADL therefore requires further distinction from both the Barthel Index and MoCA. Unlike the Barthel Index, IADL reflects more complex functional capacity required for independent community living, such as medication management, transportation, shopping, financial management, and household activities. These tasks are cognitively demanding and depend not only on global cognitive performance, but also on executive function, attention, planning ability, physical endurance, emotional status, environmental accessibility, and family support. Therefore, IADL should not be regarded as a simple downstream consequence of MoCA. A patient may show improvement in MoCA scores while still experiencing limitations in complex real-world activities, especially when executive function, confidence, endurance, or environmental adaptation remains insufficient.

In this study, approximately 70% of patients were classified into trajectories characterized by cognitive improvement, and IADL also showed longitudinal improvement when interpreted according to the scoring direction used in this study, whereby lower scores indicate better instrumental functional status. However, IADL changes did not differ significantly among the three sleep–cognition trajectory groups. This finding suggests that cognitive recovery and instrumental functional recovery may improve in parallel at the overall cohort level, but they are not interchangeable outcomes and do not necessarily show the same between-trajectory differentiation.

From a clinical perspective, these findings indicate that post-stroke follow-up should not rely solely on MoCA, NIHSS, or the Barthel Index. Patients with apparent cognitive or basic functional improvement may still require targeted assessment of instrumental functional capacity, especially in tasks such as medication adherence, transportation, financial management, shopping, and household activities. Future post-stroke management should integrate cognitive screening with IADL-oriented functional assessment to identify patients who appear to recover on conventional scales but remain vulnerable in independent community living.

The trajectory findings can be translated into three differentiated follow-up priorities. For patients in the sleep improvement–cognitive decline trajectory, the main concern is hidden cognitive deterioration despite apparent improvement in sleep. These patients should receive repeated cognitive screening, assessment of medication management ability, evaluation of instrumental activities of daily living, and family education regarding early signs of memory, attention, and executive dysfunction. For patients in the sleep decline–cognitive improvement trajectory, the priority is not additional cognitive screening alone, but active identification and management of worsening sleep problems. Follow-up should assess sleep rhythm, daytime activity, pain, nocturia, medication effects, depressive symptoms, caregiving stress, and symptoms suggestive of sleep-disordered breathing. For patients in the sleep improvement–cognitive improvement trajectory, the overall pattern is favorable, but the higher PHQ-9 scores observed in C3 at early follow-up indicate that emotional symptoms should still be monitored. Therefore, trajectory classification should not be used merely to label patients; it should be used to determine what should be monitored most closely after discharge: cognition in C1, sleep in C2, and emotional status in C3.

In conclusion, AIS survivors showed heterogeneous and partly asynchronous sleep–cognition recovery trajectories during the first year after discharge. The main clinical implication is that post-stroke recovery should not be judged by neurological function, basic activities of daily living, cognition, or sleep alone. Improvement in one domain may coexist with deterioration in another. Therefore, sleep quality and cognitive function should be monitored in parallel throughout post-discharge follow-up. The three trajectory patterns provide clinically meaningful follow-up priorities: cognitive surveillance for patients with sleep improvement but cognitive decline, sleep-focused assessment for patients with cognitive improvement but sleep deterioration, and emotional monitoring even among patients with simultaneous sleep and cognitive improvement. These findings support a more targeted post-stroke follow-up model integrating sleep, cognition, mood, and instrumental functional capacity.

## Data Availability

The raw data supporting the conclusions of this article will be made available by the authors, without undue reservation.
